# Production of Biosurfactants by Ascomycetes

**DOI:** 10.1155/2021/6669263

**Published:** 2021-04-14

**Authors:** Michele Alves Sanches, Isabella Galvão Luzeiro, Ana Cláudia Alves Cortez, Érica Simplício de Souza, Patrícia Melchionna Albuquerque, Harish Kumar Chopra, João Vicente Braga de Souza

**Affiliations:** ^1^Program in Biodiversity and Biotechnology of the Bionorte Network (PPG-BIONORTE), Amazonas State University (UEA), Manaus, Brazil; ^2^INPA Scientific Initiation Program, National Institute for Amazonian Research (INPA), Manaus, Brazil; ^3^Mycology Laboratory, National Institute for Amazonian Research (INPA), Manaus, Brazil; ^4^Higher School of Technology, Amazonas State University (UEA), Manaus, Brazil; ^5^Department of Chemistry, Sant Longowal Institute of Engineering and Technology, Sangrur 148106, Longowal, Punjab, India

## Abstract

Surfactants are utilized to reduce surface tension in aqueous and nonaqueous systems. Currently, most synthetic surfactants are derived from petroleum. However, these surfactants are usually highly toxic and are poorly degraded by microorganisms. To overcome these problems associated with synthetic surfactants, the production of microbial surfactants (called biosurfactants) has been studied in recent years. Most studies investigating the production of biosurfactants have been associated mainly with bacteria and yeasts; however, there is emerging evidence that those derived from fungi are promising. The filamentous fungi ascomycetes have been studied for the production of biosurfactants from renewable substrates. However, the yield of biosurfactants by ascomycetes depends on several factors, such as the species, nutritional sources, and environmental conditions. In this review, we explored the production, chemical characterization, and application of biosurfactants by ascomycetes.

## 1. Introduction

Biosurfactants are compounds that are produced by plants and animals, but are largely produced by microorganisms, such as bacteria, yeasts, and filamentous fungi. The combination of several properties including biomolecules (proteins, carbohydrates, and lipids) reduces surface tension to act as an an emulsifier [1]. All biosurfactants are amphiphilic and consist of polar and nonpolar parts.

The demand for biosurfactants has increased because of their environmental compatibility and versatility in emulsification. Other advantages of biosurfactants include biodegradability, low toxicity, and tolerance to different environmental factors (pH, temperature, and salinity). Given these advantages, the interest by the scientific community in studying the potential of biosurfactants has increased substantially [2]. However, issues such as high production costs and difficulties in the recovery of the pure products must be addressed before large-scale production at an industrial level can commence, allowing for their synthetic counterparts to have a competitive advantage [3, 4]. In addition, the combination of microorganisms and culture media used for production directly influences the recovery of biosurfactants during downstream processes.

The cost of biosurfactant production and recovery are the limiting factors in the industrial production of these molecules, as indicated by several recent studies [5–7]. In contrast, several studies have demonstrated laboratory-scale alternatives as a probable solution to the challenge involving production [2, 8].

The structural diversity of biosurfactants enables various applications in many industries such as food, pharmaceuticals, and cosmetics. Future industrial biosurfactant production depends on the ratio between production costs and application benefits [9, 10]. Therefore, the optimization of physicochemical and nutritional parameters and characteristics of biosurfactants needs to be studied for the development at an industrial scale [3]. In recent years, researchers have largely focused on the production of rhamnolipid and sophorolipid biosurfactants from bacteria and yeast, whereas the commercial utilization of fungi for biosurfactant production has been very limited and, thus, only few reports are available on the subject [8]. Therefore, the main focus of this review was to discuss strategic tools for the enhancement of biosurfactant production from the fungi ascomycetes and their applications.

## 2. Synthetic Surfactants vs. Biosurfactants

**Figure 1:**
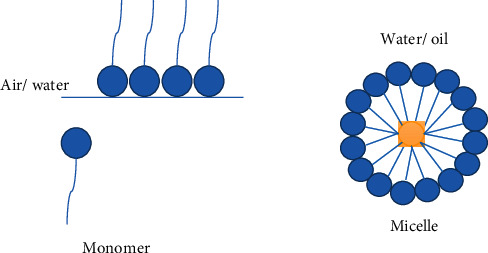
Interfacial and surface tension of the surfactant monomer (polar head and hydrocarbon tail); micelle formation in water/oil.

Surfactants are versatile molecules composed of two distinct polar and nonpolar components [11]. They act at the interface between liquids at different polarities by reducing surface tension and producing emulsions [12] (Figure 1).

**Figure 2:**
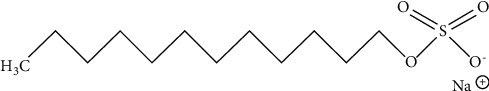
Chemical structure of sodium dodecyl sulfate.

Synthetic and chemical surfactants are conventionally produced by organic functional group transformation reactions of petroleum-based raw materials [13]. Based on the charge of their hydrophilic head, surfactants are classified as ionic, (negatively charged), cationic (positively charged), nonanionic (without any charge), or amphoteric (both positively and negatively charged); the hydrophobic tail of the surfactant is characterized by a long chain of fatty acids. The most commonly used commercial surfactants are sodium lauryl sulfate (sodium dodecyl sulfate) (Figure 2) and ammonium lauryl sulfate, which are used in the cleaning and cosmetic industries [11]. Domestic and industrial consumption of surfactants has increased in recent years without limits and restrictions. Accumulation of these surfactants in the ecosystem can lead to environmental problems [14], in addition to the manifestations of surfactant toxicity [10, 14–16]. Synthetic surfactants can reach toxic levels when they exceed the concentrations than those prescribed, in terms of hydrophobicity and chemical structure characteristics [3, 14]. Although there have been a large number of reports in the literature about the adverse effects of surfactants on the environment and human health, a total ban on the use of surfactants is impossible due to the lack of economically viable alternatives. Considering these, the utilization of biosurfactants can decrease the use of synthetic surfactants [10, 14].

**Figure 3:**
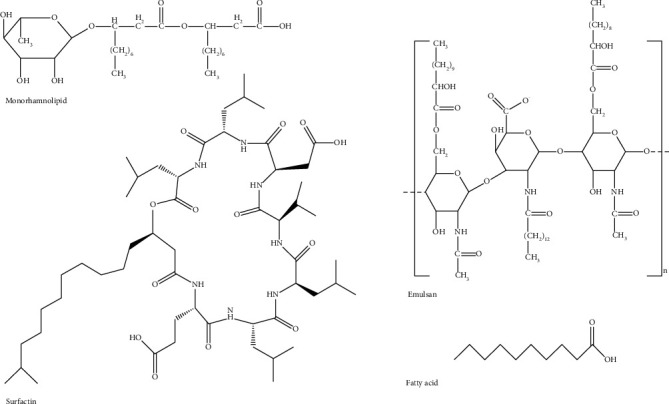
Structural diversity of some common biosurfactants. *∗*Structures are designed with the aid of the Chemdraw program.

Biosurfactants are natural surfactants synthesized by plants (e.g., saponin), animals (e.g., phospholipids, pulmonary surfactants, and bile salts), and microorganisms (e.g., glycolipids). Biosurfactants derived from microbes exhibit surfactant properties as they decrease surface tension and have high emulsifying capacity [11]. However, these biosurfactants are structurally more complex than synthetic surfactants as they are formed from combinations of biomolecules (proteins, carbohydrates, and lipids). Biosurfactants from microorganisms are classified based on their chemical structure, e.g., glycolipids are composed of carbohydrates (glucose, rhamnose, and galactose) combined with long-chain aliphatic acids or hydroxyaliphatic acids (fatty acids containing hydroxyl (OH) groups and alkyl branches); lipopeptides are formed from biomolecules in which the amino acids are bonded to carboxyl and hydroxyl groups of a 14-carbon fatty-acid chain; and polymeric biosurfactants are polysaccharide-protein complexes [11, 17–19] (Figure 3).

Biosurfactants show better properties than their synthetic counterparts. Some of the important properties observed in most biosurfactants are given below:

- Low toxicity: given that biosurfactants are used in cleaning, food and cosmetic products, and in bioremediation, determining that biosurfactants indeed have low or no toxicity is essential. Recent studies have demonstrated the absence of toxic effects by biosurfactants against microorganisms or microcrustaceans or in the germination of seeds; the potential of biosurfactant use in bioremediation of contaminated soil and water has also been demonstrated [20]. Tests to check for the toxic application of surfactants in detergents include acute dermal irritation, acute oral toxicity (LD_50_ and LC_50_), surface activity, washing efficiency, and compatibility tests with purified hard water [21].
- High biodegradability: biosurfactants are degradable in water and soil, which allows them to be used in the process of bioremediation, to release contaminants from soil, in pesticide formulations, and in biological control [22–24].
- Tolerance to pH variation, salinity, and temperature: biosurfactants have gained increased research attention for their commercial application owing to the novel biosurfactants being able perform efficiently under extreme temperatures, pH, and salinity [3, 25].
- Use of renewable substrates: the use of economically cheaper substrates renders a cost-effective biosurfactant production process in industries [6].
- Widespread applications: various types of biosurfactants have shown potential for application in numerous areas, owing to their emulsifying, antimicrobial, antitumor, antiadhesive, and anticorrosion activities. These properties are of interest to the food, textile, and biomedical industries.

The foaming properties of biosurfactants in comparison to that of synthetic surfactants make them a promising alternative for commercial production using raw materials [26, 27]. However, there are limitations associated with the production of biosurfactants, and strategies must be adopted to ensure that they can be competitive against synthetic surfactants. These strategies include the use of renewable substrate residues to reduce initial production costs and the development of efficient bioprocesses including optimization of culture conditions, improvement of downstream processes, and the use of improved strains with genetic modifications or of naturally productive potential [26].

Rhamnolipids and sophorolipids have been studied extensively and have consequently gained prominence in their applications in the global industrial sector [28]. The biotechnological advancements in the production of biosurfactants on an industrial scale are closely monitored by concerned industries. Among these, Evonik Industries pioneered the production of key components for the production of shampoos, shower gels, and household cleaning products [29]; such biosurfactants are produced mainly by bacteria and yeasts, although no biosurfactants produced by filamentous fungi are commercially available [30–32]. However, several studies have demonstrated the potential of fungi as producers of tension-active molecules [33] and with even greater yields in comparison to biosurfactants produced from bacteria [34].

## 3. Biosurfactant Production by Ascomycetes

Ascomycetes are a type of asexual fungi, or anamorph, which produce asexual spores (such as conidia) on branching structures called conidiophores. They are septate fungi with filaments partitioned by cells called septa [35, 36]. Ascomycetes biosynthesize secondary metabolites by absorption or exchange (heterotrophy). As they represent the largest group of fungi, 57,000 known species from approximately 6100 genera of ascomycetes exist in a variety of forms, including molds, yeasts, or sporocarps, and the term ascoma is generally used to describe their “fleshy” fruiting body [37, 38].

Ascomycetes are abundant in the soil, but can also be found in aquatic environments and in plants as several of them are phytopathogens [39, 40]. Egidi et al. [40] identified patterns and ecological drivers of dominant soil fungal taxa occurring in the Ascomycota phylum. Studies indicate that globally distributed fungi include genera such as *Alternaria*, *Aureobasidium*, *Cladosporium, Penicillium*, *Fusarium*, *Chaetomium*, *Acremonium*, and *Curvularia*, which are anemophilous fungi that can be dispersed through air. Most of these dominant fungi are characterized by their genomic potential for use in biotechnological resources, competition between microorganisms in the environment, and stress tolerance compared to other fungi; however, studies suggest that ascomycetes may be better equipped to withstand environmental stresses and can utilize a higher number of resources, thus leading to more generalist strategies that may contribute to their increased dominance in soils.

A*spergillus*, *Penicillium*, *and Fusarium*, among others, are ascomycetes that are being studied as biosurfactant producers [41–43].  Table 1 provides an overview of a few sources of isolation and types of reported biosurfactants produced by these ascomycetes. It can be concluded that *Aspergillus*, *Penicillium*, and *Fusarium* are the genera most studied for the production of biosurfactants. Filamentous ascomycetes showed advantages in the production of biosurfactants [66] and displayed a potential to produce biosurfactants with higher yields in comparison to those by yeasts [67]. Therefore, the abovementioned genera are promising producers of biosurfactants and emulsifiers with stable emulsions and have an excellent capacity to reduce surface and interfacial tension [44, 49, 53, 55, 59, 68].

Ascomycetes that produce biosurfactants have been obtained from different sources such as plants, soil, and contaminated environments and can produce a variety of biosurfactants in synthetic media or on renewable substrates (Table 1). Additionally, they can be isolated from areas contaminated with oil, effluents, or hydrocarbons [45, 53, 59, 69], which increases the chances of isolating a fungus that produces the compound. However, several studies have demonstrated the isolation of areas such as soil, plants, and in marine environments [42, 49, 50, 61].

Table 1 provides an overview of the genera most commonly used for the production of biosurfactants and commonly isolated biosurfactants containing glycolipids, lipopeptides, enamides, etc. The main types of biosurfactants produced by ascomycetes are low-molecular-weight biosurfactants (such as glycolipids and lipopeptides) [70]. Glycolipids are composed of glycosyl and lipid fractions with amphiphilic properties, conferring them surfactant properties. Fungal glycolipids are intracellular metabolites [44] or secondary metabolites that help in the predominance of fungi in competition with other microorganisms in a given environmental niche [68, 71].

Lipopeptides and glycolipids are biosurfactants excreted by fungal strains produced during fermentation on mineral media with olive oil as a carbon source [44] and have higher emulsification activity than that does Triton X-100 among other chemically synthesized surfactants [46].

## 4. Biosynthesis of Biosurfactants

Biosurfactants have been synthesized using a variety of substrates. They can be produced spontaneously or induced by the presence of lipophilic compounds, pH variations, agitation speed, stresses, and low concentrations of nitrogen [72]. The first reported biosurfactants were rhamnolipids, produced by *Pseudomonas aeruginosa,* and lipopeptide (surfactin), produced by *Bacillus subtilis.* Studies looking into the production of fungi-derived biosurfactants have found the lipid mannosylethitritol (MEL)—derived from *Candida* [73].

**Figure 4:**
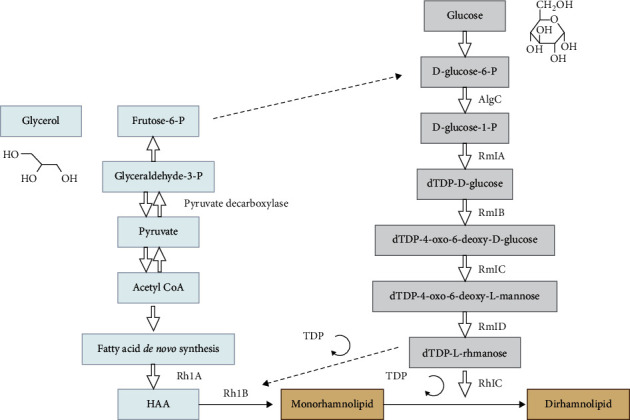
Biosynthesis related to the production of biosurfactants or bioemulsifiers using hydrophilic and hydrophobic sources as substrates [30]. ^*∗*^Image was created using Biorender.

The biosynthesis of biosurfactants generally involves separate pathways to form hydrophilic and hydrophobic moieties, which are subsequently combined [74]. The main metabolic pathways involved in the precursor synthesis of biosurfactants depend on the carbon source; the main carbon sources are carbohydrates and lipids or hydrocarbons. When carbohydrates are used as the sole carbon source in the culture medium for the production of glycolipids, carbon flow is directed to both the lipogenic (lipid formation) and glycolytic pathways (hydrophilic portion formation). In contrast, when a hydrocarbon source is used, biosynthesis is directed to the lipolytic and gluconeogenesis pathways [75]. An example of biosurfactant biosynthesis is the production of rhamnolipids by bacteria in a medium containing glycerol [30]. The molecular biosynthetic regulation of rhamnolipid, a glycolipid-type biosurfactant produced by *P. aeruginosa*, was the first to be deciphered [76] (Figure 4).

Although filamentous fungi can produce biosurfactants and it is possible to identify the type of biosurfactant, the biosynthesis of these compounds, their genetic basis, and the production route of the biosurfactant are not yet fully understood. The biosynthesis of most biosurfactants is strictly regulated, and the biosynthesis of hydrophobins by *Trichoderma reesei* depends on *hfb*1 and *hfb*2 genes [76].

An area of study that needs to be further explored is the availability of sequencing methods and tools for bioinformatic analysis that may allow the deduction of biosynthetic genes from the fungal genome biosurfactant.

## 5. Influence of Bioprocess and Nutritional Conditions on the Production of Biosurfactants

During the production process of biosurfactants, critical factors that directly influence cultivation conditions were evaluated and classified as external factors; these include agitation, aeration, and volume. Additionally, nutritional factors are directly related to the microorganism used and therefore influences the type of biosurfactant produced [67]. The synthesis of biosurfactants depends largely on the availability of carbon sources and the balance between carbon and other nutrients. Each fungus has specific nutritional needs that favor the production of a class of biosurfactants, but this optimization/characterization correlation is yet to be explored, with an increase in the yield of biosurfactant production being the main objective of this field of research (Table 2).

Carbon sources play an important role in the growth and production of biosurfactants by various microorganisms and vary from species to species. The main sources of carbon used for the production of ascomycetes are soybean oil, crude oil, agroindustrial residues, hydrocarbons, and glucose [50, 57, 58, 60, 61, 78].

Nitrogen is the second most important supplement for the production of biosurfactants by microorganisms. Various organic and inorganic nitrogen sources have been used in the production of biosurfactants as a mineral medium, yeast extract, and peptone [57, 58, 60, 61, 67, 79]. Supplementation with glucose as a carbon source can increase biosurfactant production, and yeast extract as a nitrogen source also plays an important role in biosurfactant production [50] (Table 2).

The important characteristics of most organisms are their strong dependence on the pH for cell growth and for the production of secondary metabolites. They produce the highest yield of biosurfactants in the range of pH 6–7 [50, 61, 78]. Biosurfactant composition depends on the fungal isolation conditions: a fungus isolated from marine environment requires the addition of salt in the culture medium. If the organism was isolated from a place contaminated with oil, oil can be used as an inducer in the bioprocess [45, 50, 59].

Most bioprocess conditions occur in incubation temperatures between 25–30°C and agitation between 100 and 150 rpm over around 3–20 days [57–59, 61]. These conditions can be optimized and customized for each fungus, such as by changing the appropriate pH and nutrient composition (Table 2).

Statistical analyses of cultivation conditions along with factorial design have proven to be effective tools for optimizing the production of biosurfactants. Such tools result in a reduction of the number of laboratory experiments, acquisition of mathematical models that can be used in industry, demonstrating which factors are important, and their interaction in the production of biosurfactants. Table 2 provides an overview of some optimization processes and conditions for the growth of certain ascomycete fungi. The search for the best conditions (such as substrate, pH, and temperature) for biosurfactant production has been explored, specifically in the use of agroindustrial waste [50, 52, 53].

The use of statistical tools is very efficient in increasing the production and properties of biosurfactants, most of which are factorial designs and response surface methodologies (RSMs), with the objective of optimizing the response, which is influenced by several independent variables [50, 61, 80]. The association of filamentous fungi producing biosurfactants has been extensively studied using these statistical tools; in the last few years, the use of Plackett–Burman as a selection tool stands out, considering that authors test various fungi, conditions, and variables [59].

Several factors, such as availability of carbon and nitrogen sources, pH, agitation, and incubation, need to be optimized to enhance biosurfactant yield. Table 2 summarizes the data obtained from studies that determined a high emulsification index (>60%) and a reduction in surface tension (<25 mN/m) through optimizations for the production of biosurfactants by ascomycete fungi.

In general, the use of statistical approaches to determine the effect on the factors analyzed and their interactions result in the enhancement of production biosurfactant yields, and the possibility of reproducing the mathematical model provides information of interest to industries.

## 6. Extraction, Purification, and Chemical Characterization of Biosurfactants

After the production of biosurfactants, the next important step is to recover them from the fermentation media followed by purification to make them readily available for various industrial applications [67]. In the bioprocess of obtaining the biosurfactant, the extract is still limited because of the cost of recovery, purification processing, or both; the cost is approximately 60% of that of the total production [6].

Methods to reduce production costs have been studied, mainly using renewable resources; however, these resources contaminate or hinder the extraction and purification process and, consequently, the characterization of biosurfactants. Several techniques are used to obtain products of biotechnological interest, such as recovery with water-miscible solvents, such as acetone and ethanol, acidification, and the addition of salts to the solution.  Table 3 shows the recovery methods for biosurfactants produced by filamentous fungi. The main methods used were acidification, precipitation with alcohol, and solvents.

Following the characterization process, the crude biosurfactants are analyzed using a combination of spectroscopic techniques such as mass spectrometry, infrared (IR), and nuclear magnetic resonance (NMR) spectroscopy to elucidate the structure of the biosurfactant. Chromatographic techniques such as gas chromatography (GC) and high-performance liquid chromatography (HPLC) in combination with mass spectrometry (MS) are also used for the characterization and purification of biosurfactants. In the investigation of the biosurfactant produced by *Fusarium fujikuroi*, the compounds were identified by gas chromatography coupled with MS and with a flame ionization detector [59].

Fourier transform infrared spectroscopy is often used to identify organic functional groups (alkyl, carbonyl, ether, and ester linkages in carbohydrates). In a study carried out by Pele et al., [89] the infrared spectra suggested the presence of an ester linkage, and an amide group confirmed the presence of glycoproteins on the structure of a biosurfactant produced by *Rhizopus arrhizus* UCP 1607.

The characterization of the biosurfactants produced by filamentous fungi is still scarce, and it, at times, becomes necessary to use other techniques such as matrix-assisted laser desorption/ionization-time-of-flight MS (MALDI-TOF MS).

According to Table 3, the primary process of extracting biosurfactants by filamentous fungi is precipitation and solvent fraction. Precipitation can be promoted by acidification (HCl) or utilization of solvent systems such as chloroform, methanol, ethyl acetate, and ethanol. In addition, previous studies have demonstrated that combinations of analytical methodologies are necessary for chemical characterization, and the techniques to identify biosurfactants include thin-layer chromatography (TLC), HPLC, Fourier transform infrared (FT-IR), and MS.

## 7. Industrial Applications

The exploration of natural resources in the biotechnological era has promoted scientific and technological advancements by adding value to natural products. Biosurfactants have the potential for the development of significant biotechnological processes in the 21^st^ century owing to their unique emulsification properties [7].

Biosurfactants may be used as therapeutic agents because of their antibacterial, antifungal, and antiviral properties [90, 91]. These molecules have ideal properties for incorporation in food and cosmetic formulations, and their antimicrobial and antibiofilm potential are of great interest to food processing industries [92]. Studies have investigated and established the promising potential applications of biosurfactants (some fungi have also been deposited in the patent bank; Table 4) as an alternative to synthetic surfactants in the industry. The biosurfactant application market is segmented into the food, cosmetics, health, and textile industries, with household detergents and personal-care products as the main applications of biosurfactants.

Most biosurfactant industries are in North America, Asia, and Europe; consequently, they are the main consumers. Europe dominated the biosurfactant market with a global share of 52.5% in 2019. This is attributable to the increasing awareness among consumers regarding the health hazards associated with chemical surfactants [2]. However, Latin America has immense potential owing to its enormous biodiversity and several agroindustrial residues produced that can be used as substrates for biosurfactant production; thus, the development of biosurfactants in South America, specifically in Brazil, remains a challenge, although Brazil is a leader among Latin American countries in biosurfactant research, with a high number of articles and patents [93].

The production of biosurfactants by ascomycetes filamentous fungi has been increased in recent years. Among the ascomycetes, some fungi will stand out in the industrial sector because they have demonstrated production potential when grown using different synthetic culture media and renewable substrates, in addition to their biological activities. Biosurfactants obtained from fungi have a variety of chemical classes, which favor several applications across industrial, food, biomedical, and environmental sectors. There remains a need for further studies on the patents, characterization, and application of these substances. Challenges concerning the understanding of metabolic production pathways, genes of interest, and techniques for the recovery of biosurfactants remain and should be addressed in future studies.

## 8. Conclusions

- Ascomycetes isolated from various environments have the potential to produce biosurfactants using renewable substrates.
- The main genera of ascomycetes that are able to produce biosurfactants include species from *Aspergillus*, *Fusarium,* and *Penicillium*, which mainly produce glycolipids and lipopeptides.
- The production of biosurfactants from ascomycetes is largely affected by various differences in growing media conditions, pH, temperature, and carbon and nitrogen sources. The influence of production factors has been studied, mainly through design factorial and response surface methodologies.
- The main extraction methods to obtain biosurfactants from culture media include acidification or the use of a solvent system, and characterization and structure elucidation of the biosurfactants have been achieved using a combination of analytical, chromatographic, and spectroscopic techniques.

The use of biosurfactants obtained by ascomycetes needs to be encouraged, given the advantages in comparison to their synthetic counterparts, data from several studies, and their potential to produce ecologically safe and sustainable products.

## Figures and Tables

**Table 1:** Several classes of biosurfactants produced by fungi isolated from different environmental sources.

Fungi	Source	Type of biosurfactant	Reference
*Aspergillus niger*	—	Monoglucosyloxyoctadecenoic (glycolipid)	[44]
*Cladosporium resinae*	Jet fuel	Cladosan	[45]
*Curvularia lunata* IM 2901	Collection	Polymeric biosurfactant	[46]
*Penicillium citrinum*	—	Glycolipid	[47]
*Penicillium* sp.	Soil	—	[48]
*Aspergillus fumigatus*	Soil	—	[49]
*Aspergillus ustus* MSF3	Marine sponge (*Fasciospongia cavernosa*)	Glycoprotein	[50]
*Exophiala dermatitidis* SK80	Soil	Monoglycerides	[51]
*Aspergillus niger*	Soil	Glycolipid	[52]
*Aspergillus flavus*	Soil contaminated with diesel oil	—	[53]
*Fusarium* sp.	Plant (*Melia azedarach*)	Fusaroside (glycolipid)	[54]
*Aureobasidium pullulans*	Plant (*Lilium lancifolium*)	L9	[55]
*Fusarium* SP BS-8	Soil	Lipopeptide	[56]
*Penicillium chrysogenum SNP5*	Soil	Lipopeptide	[57]
*Fusarium proliferatum*	Rice bran	Enamide	[58]
*Fusarium fujikuroi*	Soil contaminated with hydrocarbons	*α*, *β*-Trehalose (glycolipid)	[59]
*Xylaria regalis*	Plant (*Thuja plicata*)	—	[60]
*Fusarium oxysporum* LM5634	Soil	—	[61]
*Fusarium* sp.	Collection	Glycolipid	[62]
*Aspergillus niger*	Plant (*Piper hispidum*)	—	[63]
*Penicillium chrysogenum* MUT 5039	Marine	Sap-Pc protein	[64]
*Aspergillus terreus* MUT 271	Marine	Cerato-platanins	[65]

**Table 2:** Different statistical strategies used for the optimization of biosurfactant production by ascomycetes.

Fungi	Type of biosurfactant	Process optimization	Optimized condition	*E*_24_ (%)/ST (mN/m)	Reference
*Aspergillus ustus* (MSF3)	Glycoprotein	pH, temperature, salt concentration, carbon nitrogen sources, and metals (univariate and response surface methodology)	pH 7/3% NaCl/glucose and cheapest raw/ratio C : N 3 : 2	75%	[77]
*Penicillium* 8CC2	—	Sources of carbon and nitrogen, pH, and production time using factorial design with repetition at the central point	Soybean oil, 20 g/L^−1^; yeast extract, 30 g/L^−1^; pH6	79.82%	[42]
*Fusarium fujikuroi*	*α*, *β*-Trehalose	pH, incubation time, agitation, and inoculum (Plackett–Burman + central rotational compound arrangement)	Temperature, agitation, and incubation time variables, significantly temperature 47 ◦C, 120 rpm for 7 days of incubation	PB: 24.08 mN/m	[59]
				CCRD: 20.08 mN/m	
*Fusarium oxysporum*	—	Agroindustrial substrate, carbon source, nitrogen, pH, and agitation factorial design	Waste significantly influenced	67.74%	[61]

^*∗*^ *E*_24_ (%)- emulsification index, ^*∗∗*^ST- surface tension.

**Table 3:** Types of biosurfactant extraction and characterization from filamentous fungi.

Fungi	Process/solvent used in the purification	Analytical method	Reference
*Cladosporium resinae*	Reverse-phase chromatography	High-performance liquid chromatography (HPLC)	[45]
*Curvularia lunata*	Acetone/lyophilized	Gas chromatography (GC)	[46]
*Penicillium* sp.	Cold acetone 4°C	HPLC	[48]
*Aspergillus fumigatus*	Distilled water 90°C and solvent	Emulsifying activity	[49]
*Aspergillus* sp. O-4	Phosphate buffer pH 7.0/0.2 M/distilled water at 90°C	Emulsifying activity and surface tension	[81]
*Exophiala dermatitidis SK80*	Ethyl acetate	Thin-layer chromatography (TLC)	[51]
*Aspergillus* sp. MSF1	Solvents	TLC, Fourier transform infrared (FT-IR), and HPLC	[79]
*Aspergillus niger*	Acid precipitate HCl/chloroform and methanol	TLC	[52]
*Penicillium chrysogenum* SNP5	Ethanol −20 ºC	TLC and FT-IR	[82]
*Aspergillus Niger* and *Aspergillus flavus*	Acid precipitation with 1 M of H_2_SO4 pH 2.0/chloroform and methanol	Gas chromatography-mass spectrometry (GC-MS)	[78]
*Aspergillus flavus*	Ethyl acetate	Infrared spectroscopy (IR) and mass spectrometry (MS)	[83]
*Pleurotus ostreatus*	Chloroform:ethanol	FT-IR	[84]
*Fusarium proliferatum*	Acidification pH 2.0 HCl 6 N/solvent system	TLC-column chromatography, FT-IR, and nuclear magnetic resonance spectrometry (NMR)	[58]
*Pleurotus djamor*	HCl pH 2.0 acidification	FT-IR	[85]
*Pleurotus sajor-caju*	Acidification by HCl 6 N pH 2.0/isopropanol	FT-IR	[86]
*Ceriporia lacerate*	Ethyl acetate	LC-MS and GC-MS	[87]
*Fusarium oxysporum*	Solvent chloroform:methanol	FT-IR, NMR-spectral studies, and GC	[88]
8CC2 *Penicillium*	Precipitation with ethanol	Stability studies	[42]
*Fusarium fujikuroi*	Acidification with 6 M HCl pH 4.0/solvent acetate:methanol (1 : 4)	Nuclear magnetic resonance spectrometry (NMR)	[59]
*Xylaria regalis*	Acidification pH 2.0 HCl (1 N)/ethyl acetate	High-performance thin-layer chromatography (HPTLC)	[60]
*Aspergillus terreus* MUT 271 and *Trichoderma harzianum* MUT 290	Concentrated by air bubbling and by an Amicon Ultrafiltration cell<	Q-TOF LC/MS and circular dichroism spectroscopy	[65]

**Table 4:** Filamentous fungi producing biosurfactants deposited in the patent bank.

Microorganism	Title	Inventor and date	Patent
*Cladosporium resinae*	Preparation of a new biosurfactant	Jimenez and Morales (1993)	ES 2039187B1
Fungi	Method for preparing rhamnolipid	Yang (2007)	CN 1891831
*Aureobasidium pullulans*	New biosurfactant produced by *Aureobasidium pullulans*	Kim et al. (2013)	KR 101225110
*Trichoderma*	Hydrophobin production by *Trichoderma*	Quay (2002)	US7863245B2

## Data Availability

All data generated or analyzed during this study are included within the article.
